# Aggressive blood pressure reduction is not associated with decreased perfusion in leukoaraiosis regions in acute intracerebral hemorrhage patients

**DOI:** 10.1371/journal.pone.0213645

**Published:** 2019-03-11

**Authors:** Mahesh Kate, Laura Gioia, Thomas Jeerakathil, Michael D. Hill, Bronwen Gould, Rebecca McCourt, Dar Dowlatshahi, Shelagh Coutts, Jayme Kosior, Andrew Demchuk, Brian Buck, Kenneth Butcher

**Affiliations:** 1 Division of Neurology, Department of Medicine, University of Alberta, Edmonton, Canada; 2 University of Calgary, Calgary, Canada; 3 University of Ottawa, Ottawa, Canada; 4 Prince of Wales Clinical School, University of New South Wales, Sydney, Australia; University of Glasgow, UNITED KINGDOM

## Abstract

Leukoaraiosis regions may be more vulnerable to decreases in cerebral perfusion. We aimed to assess perfusion in leukoaraiosis regions in acute intracerebral hemorrhage (ICH) patients. We tested the hypothesis that aggressive acute BP reduction in ICH patients is associated with hypoperfusion in areas of leukoaraiosis. In the ICH Acutely Decreasing Arterial Pressure Trial (ICH ADAPT), patients with ICH <24 hours duration were randomized to two systolic BP (SBP) target groups (<150 mmHg vs. <180 mmHg). Computed tomography perfusion (CTP) imaging was performed 2h post-randomization. Leukoaraiosis tissue volumes were planimetrically measured using semi-automated threshold techniques on the acute non-contrast CT. CTP source leukoaraiosis region-of-interest object maps were co-registered with CTP post-processed maps to assess cerebral perfusion in these areas. Seventy-one patients were included with a mean age of 69±11.4 years, 52 of whom had leukoaraiosis. The mean relative Tmax (rTmax) of leukoaraiotic tissue (2.3±2s) was prolonged compared to that of normal appearing white matter in patients without leukoaraiosis (1.1±1.2s, *p* = 0.04). In the 52 patients with leukoaraiosis, SBP in the aggressive treatment group (145±20.4 mmHg, n = 27) was significantly lower than that in the conservative group (159.9±13.1 mmHg, n = 25, *p* = 0.001) at the time of CTP. Despite this SBP difference, mean leukoaraiosis rTmax was similar in the two treatment groups (2.6±2.3 vs. 1.8±1.6 seconds, *p* = 0.3). Cerebral perfusion in tissue affected by leukoaraiosis is hypoperfused in acute ICH patients. Aggressive BP reduction does not appear to acutely aggravate cerebral hypoperfusion.

## Introduction

Recent studies suggest leukoaraiosis is an independent predictor of worse short and long-term outcomes in intracerebral hemorrhage (ICH), including increased early mortality rates.[1–4] The mechanisms of leukoaraiosis associated poor outcomes are unknown, but may be related to increased ICH volume and/ or expansion at presentation.[5] Most concerning, and relevant to acute blood pressure management, is the fact that sub-acute ischemic lesions appear to be associated with leukoaraiosis, raising the possibility that these patients are more hemodynamically susceptible to perfusion changes.[6]

Cerebral metabolism[7], relative cerebral blood volume (rCBV)[8], cerebral blood flow (CBF)[9] and cerebrovascular reactivity[10,11] are reduced in leukoaraiosis regions in patients without acute stroke. Whether the presence of low cerebral perfusion parameters and decreased metabolism is causally related or secondary to leukoaraiosis is unknown.[12] It has also long been postulated that patients with long-standing hypertension shift the autoregulatory curve to the right.[13] As leukoaraiosis is a putative marker of chronic hypertension, it is also possible that the perfusion response to BP reduction is altered in these patients.[14]

In the Intracerebral Hemorrhage Acutely Decreasing Arterial Pressure Trial (ICH ADAPT), we demonstrated that perihematoma hypoperfusion is unaffected by blood pressure (BP) reduction.[15] The effect of acute BP reduction on leukoaraiotic tissue perfusion in ICH is unknown. Using ICH ADAPT data, we aimed to assess perfusion in leukoaraiosis regions in acute intracerebral hemorrhage (ICH) patients. We tested the hypothesis that aggressive acute BP reduction in ICH patients is associated with hypoperfusion in areas of leukoaraiosis.

## Materials and methods

### Patients

ICH ADAPT was a multicenter randomized controlled trial of aggressive (<150 mmHg) versus conservative (<180mmHg) BP management in ICH patients. The trial protocol has been described previously (ClinicalTrials.gov Identifier: NCT00963976).[16] Human Research Ethics Office of University of Alberta, Canada approved the study. Acute ICH patients >18 years of age diagnosed with non-contrast CT (NCCT) scan within 24h of symptom onset without contraindications to CT perfusion (CTP) and at least two systolic BP readings ≥150 mm Hg were eligible. All patients or their legally acceptable surrogate signed a written informed consent form. Intravenous labetalol, enalapril and/or hydralazine were used to reduce BP to target levels within 1 hour of randomization. A CTP scan was performed two hours after randomization.

### Imaging protocol

All patients underwent a diagnostic NCCT brain scan prior to randomization. This consisted of 5 mm slices through the entire brain. Two hours after randomization, all patients underwent a repeat NCCT brain scan. A 38 to 80 mm thick section (slab thickness varied with scanner capabilities) was selected to assess perfusion, centered on the slice where the hematoma had the greatest diameter on the NCCT. Perfusion CT images were acquired with IV iodinated contrast (40 mL) given over 10 seconds, via an 18-gauge angiocatheter in an antecubital vein with CT images acquired every second for 50 seconds (80 kvp, 200 mA per image). All patients had a repeat NCCT scan at 24±3 hours.

### Image analysis

Hematoma (intraparencyhmal and intraventricular) and leukoaraiosis volumes were measured by raters blinded to BP treatment, using planimetric (semi-automated threshold) techniques on the baseline NCCT scan using the ANALYZE 11.0 software package (Biomedical Imaging Resource, Mayo Clinic, Overland Park, KS, USA).[17] A Hounsfield Unit (HU) threshold was used to define leukoaraiosis within the periventricular white matter regions. Only Voxels between 18HU and 34HU were included for analysis. Areas of previous infarcts (<18HU voxels), Cerebrospinal fluid, Virchow Robin spaces and perihematoma edema were excluded. The distribution and extent of the leukoaraiosis region-of-interest maps generated by the threshold method were also used for qualitative grading using the Fazekas scale.[18] Perihematoma edema volume was measured on the 24h NCCT scan, using planimetric techniques and threshold of <24HU.[19]

**Fig 1:**
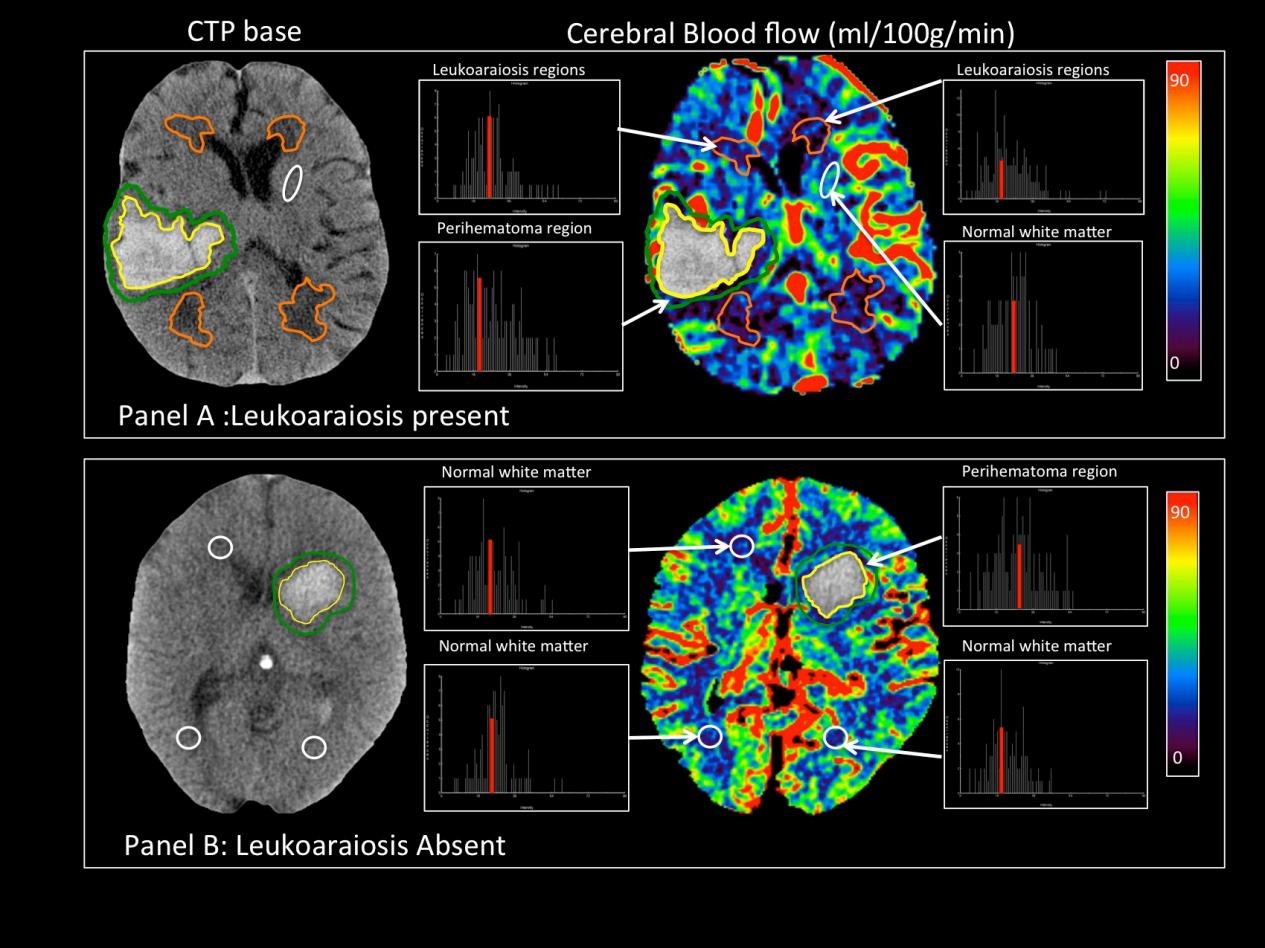
**Measurement of perfusion parameters in patients with leukoaraiosis (A) and without leukoaraiosis (B).** A: Regions-of-interest in the perihematoma region (green), leukoaraiosis tissue (orange) and normal white matter (white). B: Region-of-interest object maps were drawn Perihematoma region (green), normal white matter (white). The histograms in demonstrate the distribution of the absolute cerebral perfusion values in the object maps.

CTP source images were post-processed using a semi-automated (PerfScape, Olea Medical, Marseilles, France) after manual selection of an arterial-input-function using the singular value decomposition (SVD) deconvolution method.[20] Processed perfusion maps cerebral blood flow (CBF), cerebral blood volume (CBV), time-to-peak of the impulse response (Tmax) and mean-transit-time maps (MTT) were transferred to ANALYZE 11.0 for region-of-interest analysis. Mean perfusion parameters were calculated in periventricular leukoaraiotic regions **(Fig 1**) by co-registered region-of-interest object maps drawn on CTP source (pre-contrast injection) images using semi-automated HU threshold technique described above. In patients without leukoaraiosis, perfusion parameters were calculated in 1cm diameter circular regions-of-interest in the anterior and posterior periventricular region of each hemisphere at two different levels (at the level of the basal ganglia and 1 cm superior to basal ganglia level) **(Fig 1**).

A 1cm diameter oval region-of-interest object map in normal appearing deep white matter contralateral to the hematoma (anterior limb of internal capsule) and four additional circular region-of-interest object maps in the centrum semiovale were used as reference white matter regions in patients with and without leukoaraiosis. Periventricular relative CBF, CBV and MTT were calculated as ratio of periventricular white matter perfusion to reference white matter region perfusion. Relative Tmax (rTmax) was defined as a difference between periventricular white matter perfusion and reference white matter region perfusion. Ischemia was defined as rTmax >4 seconds and hypoperfusion was defined as rTmax between 2–4 seconds.

### Statistical analysis

Statistical analysis was performed using SPSS 21.0 (IBM SPSS Statistics 2013, Armonk, NY, USA). Differences in the frequency of baseline characteristics in patients’ with and without leukoaraiosis were assessed with Pearson χ^2^ and Fisher’s exact tests. Mean/median differences between patients with/without leukoaraiosis were assessed with Student t-tests (age) and Mann-Whitney *U* tests (BP, NIHSS, total ICH volume, perihematoma edema volume, and Barthel Index at 90 days). One-way ANOVA followed by *post-hoc* Kruskal-Wallis tests were used to assess differences in mean rCBF, rCBV, rMTT and rTmax with respect to Fazekas grades. Leukoariaosis volumes were not normally distributed and were subsequently log transformed for linear regression analyses. There was no interaction between systolic blood pressure reduction and leukoaraiosis volume.

## Results

### Patients and leukoaraiosis

A total of 75 patients (54 (72%) male, mean±SD age 69.8±11.8 years) were randomized to either aggressive or conservative BP reduction. Seventy-one patients were included in the cerebral perfusion analysis (2 patients without CTP data and 2 patients with cerebellar hemorrhages, where CTP data was restricted to the posterior fossa were excluded). A total of 52 (73.2%) of patients had evidence of leukoaraiosis on NCCT. The median (IQR) leukoaraiosis volume in these patients was 8.5(3.2–23.5) ml. The median Fazekas grade was 2(1–3).

Patients with leukoaraiosis were older (71 ±11.2 vs. 65 ±11.6 years; 95% Confidence Interval (CI) 2.4–14.8, *p* = 0.01; Table 1). Patients with leukoaraiosis presented with higher blood pressure at baseline (188±20.5 vs. 175±22 mmHg; *p* = 0.04) and larger ICH volumes (19.3(7.8–36.5) vs. 14.1 (5.1–20.1) ml; *p* = 0.02). There was a significant correlation between the total ICH volume at baseline and edema volumes at 24 hours (*R* = 0.5, p<0.001). Leukoaraiosis volume (β = 6.8, [95%CI, 0.3–12.9], *p* = 0.03) and Fazekas grade (β = 10, [95%CI, 4.7–15.4], *p*<0.001) were independently associated with baseline ICH volume. Leukoaraiosis volumes (β = -3.2, [95% CI, (-4.9)-(-1.4)] *p* = 0.001,) and ICH volumes (β = 0.2, [95% CI, 0.1–0.3], *p* = 0.001) were independently associated with 24h perihematoma edema volume.

**Table 1:** Baseline characteristics of intracerebral hemorrhage patients with and without leukoaraiosis.

Characteristic	Leukoaraiosis Present (n = 52)	Leukoaraiosis Absent (n = 19)	p value
**Age, years, mean±SD**	71 ±11.2	65±11.6	0.01
**Gender (M: F)**	36:16	16:3	0.5
***Past Medical History***			
**Hypertension (%)**	43 (82.7)	8 (42.1)	0.03
**Coronary Artery Disease, n (%)**	8 (15.3)	1(5)	0.6
**Diabetes Mellitus, n (%)**	15(28.8)	5(26.3)	0.8
**Previous history of ICH, n (%)**	2(5.4)	2(10.5)	0.3
**Previous history of ischemic stroke, n (%)**	5 (10.9)	2(10.5)	0.5
***Clinical variables***			
**Systolic BP at baseline (mmHg, mean±SD)**	188±20.5	175±22	0.04
**Diastolic BP at baseline (mmHg, mean±SD)**	95.1±22.3	97.3±25.3	0.5
**Systolic BP at 2hours (mmHg, mean±SD)**	154 ±21.5	151±15	0.5
**Diastolic BP at 2hours (mmHg, mean±SD)**	78.7±16.8	79±13.5	0.8
**Randomized to aggressive arm of therapy n, (%)**	27 (51.9)	10 (52.6)	1
**NIHSS on admission median (IQR)**	11(7–18.5)	10 (5.1–12.8)	0.4
***Hematoma location***			
**Lobar, n (%)**	13 (25)	3 (15.7)	0.4
**Basal Ganglia, n (%)**	25 (48.1)	9 (47.4)	
**Total ICH volume (ml), median (IQR)**	19.3(7.8–36.5)	14.1 (5.1–20.1)	0.02
**Intraventricular Extension, n (%)**	20(38.5)	8 (42.1)	0.5
***Outcome Measures***			
**Hematoma expansion (>6ml), n (%)**	13 (25)	2 (10.5)	0.2
**24 Hours Perihematoma Edema (ml)** **median (IQR)**	3.5(3–9.1)	2.3 (1.2–3.9)	0.01
**90-Day MRS, median (IQR)**	3.3(1.5–5)	2(1–2)	0.02
**90-Day Mortality, n (%)**	9 (18.2)	1 (5)	0.2

M,male; F,female; ICH,Intracerebral hemorrhage; BP, blood pressure; NIHSS, National Institute of Health Stroke Scale; MRS, modified Rankin scale

### Cerebral perfusion and leukoaraiosis

Seventy-one patients were included in the cerebral perfusion analysis (2 patients without CTP data and 2 patients with cerebellar hemorrhages, where CTP data was restricted to the posterior fossa were excluded). The time from onset to CTP in patients with leukoaraiosis (12.6±7.3h) was similar to that in patients without leukoaraiosis (11.9±7.3h, *p* = 0.5). The mean rCBF of leukoaraiotic tissue (0.88±0.1) was lower compared to that of normal appearing white matter in patients without leukoaraiosis (0.94±0.1, *p* = 0.01,Table 2). The mean rTmax of leukoaraiotic tissue (2.3±2s) was prolonged compared to that of normal appearing white matter in patients without leukoaraiosis (1.1±1.2s, *p* = 0.04). There were no differences in the mean leukoaraiotic tissue perfusion between Fazekas grades 1–3 (Table 3).

**Table 2:** Cerebral perfusion characteristics in patients with and without leukoaraiosis.

Characteristics	Leukoaraiosis Present (n = 52)	Leukoaraiosis Absent (n = 19)	p value
***Cerebral Blood Flow (CBF) ml/100g/min***			
**Periventricular White Matter, rCBF, mean±SD**	0.88±0.1	0.94±0.1	0.01
**Perihematoma rCBF, mean±SD**	0.86±0.1	0.88±0.1	0.5
***Cerebral Blood Volume (CBV) ml/100g***			
**Periventricular White Matter, rCBV, mean±SD**	0.82±0.1	0.81±0.1	0.8
**Perihematoma rCBV, mean±SD**	0.9±0.1	0.9±0.1	0.8
***Tmax*, *seconds***			
**Periventricular White Matter, rTmax, mean±SD**	2.3±2	1.1±1.2	0.04
**Perihematoma rTmax, mean±SD**	2.2±1.5	1.5±1	0.05
***Mean Transit Time (MTT) Seconds***			
**Periventricular White Matter, rMTT, mean±SD**	0.97±3	0.99±0.1	0.5
**Perihematoma rMTT, mean±SD**	1±0.2	1±0.1	0.2

rCBF, relative cerebral blood flow; rCBV, relative cerebral blood volume; rTmax, relative Tmax; rMTT, relative mean transit time.

**Table 3:** Cerebral perfusion parameters in white matter regions according to leukoariaosis severity (Fazekas grade).

Characteristics	0 (n = 19)	1 (n = 23)	2 (n = 12)	3 (n = 17)	*p* value *(Grade 1–3)*
***Relative Cerebral Blood Flow (rCBF)***					
**Periventricular WM rCBF, mean±SD**	0.95±0.1	0.89 ±0.1	0.9±0.1	0.86 ±0.1	0.6
**PH rCBF, mean±SD**	0.87±0.1	0.88 ±0.1	0.88±0.1	0.83±0.1	0.3
***Relative Cerebral Blood Volume (rCBV*)**					
**Periventricular WM rCBV mean±SD**	0.82 ±0.2	0.88 ±0.2	0.77 ±0.2	0.78±0.1	0.1
**PH rCBV, mean±SD**	0.90±0.1	0.92±0.1	0.91±0.1	0.86±0.2	0.4
***Relative Tmax (rTmax)*, *seconds***					
**Periventricular WM rTmax, mean±SD**	1.1±0.6	1.3±2.5	2.6±2	3.4±3.5	0.2
**PH rTmax, mean±SD**	1.6±1	2.3±1.4	2.2±1.3	2±1.9	0.7
**Relative Mean Transit Time (rMTT), seconds**					
**Periventricular WM rMTT mean±SD**	0.99±0.1	0.92±0.2	0.99±0.5	1±0.2	0.5
**PH rMTT mean±SD**	1±0.1	0.96±0.1	1±0.2	1±0.3	0.4

WM, white matter; PH, Perihematoma region

#### Blood pressure treatment in leukoaraiosis patients

All the patients in <150 mmHg group received intervention, however in 180 mmHg group only 48% of patients received blood pressure lowering medications. In patients with leukoaraiosis both mean systolic BP (145±23 vs.161±14 mmHg, p = 0.005) and mean diastolic BP (72±13 vs. 84±16 mmHg, p = 0.006) at 2hours were lower in the 150 mmHg group compared to 180 mmHg group. The absolute difference in Systolic BP between the two groups in patients with leukoaraiosis was 16 mmHg at 2 hours post-treatment. With a fixed alpha of 5% we have 86.3 power post-hoc to reject the null hypothesis that there is no difference between groups (independent samples).

There were no differences in any perfusion parameters between patients with leukoaraiosis randomized to a target systolic blood pressure target of <150 mmHg (n = 27) or <180 mmHg (n = 25; Table 4). The rTmax in the leukoaraiosis regions 2hours after sustained blood pressure reduction was 2.6±2.3 seconds in the aggressive arm and 1.8±1.6 seconds in the conservative arm (p = 0.3). In eight patients with leukoaraiosis a pre-BP and post-BP reduction CTP was available. There was no difference in the pre and post-BP reduction periventricular white matter rCBF (0.90±0.3 vs. 0.86±0.1, p = 0.6) and rTmax (1.4±1.3 vs. 2.1±1.2, p = 0.2; Table 5).

**Table 4:** Cerebral perfusion parameters in the two treatment groups in patients with leukoaraiosis.

Characteristics	<150 mmHg (n = 27)	<180 mmHg (n = 25)	*p* value
***Cerebral Blood Flow (CBF) ml/100g/min***			
**Periventricular White Matter rCBF, mean±SD**	0.88±0.1	0.89±0.1	0.6
**Perihematoma rCBF, mean±SD**	0.84±0.1	0.88±0.1	0.2
***Cerebral Blood Volume (CBV*) *ml/100g***			
**Periventricular White Matter rCBV, mean±SD**	0.83±0.2	0.82±0.1	0.8
**Perihematoma rCBV, mean±SD**	0.89±0.1	0.91±0.2	0.4
***Tmax*, *seconds***			
**Periventricular White Matter rTmax, mean±SD**	2.6±2.3	1.8±1.6	0.3
**Perihematoma rTmax, mean±SD**	2.6±1.6	1.7±1.3	0.03
***Mean Transit Time (MTT)*, *Seconds***			
**Periventricular White Matter rMTT, mean±SD**	0.96±0.3	0.93±0.3	0.7
**Perihematoma rMTT, mean±SD**	0.98±0.1	1.02±0.2	0.4

SD; standard deviation; rCBF, relative cerebral blood flow; rCBV, relative cerebral blood volume; rTmax, relative Tmax; rMTT, relative mean transit time.

**Table 5:** Cerebral perfusion pre and post blood pressure reduction in leukoaraiosis areas and perihematoma regions (n = 8).

	Leukoaraiosis region		p value	Perihematoma region		p value
	*Pre BP reduction*	*Post BP reduction*		*Pre BP reduction*	*Post BP reduction*	
**rCBF, mean±SD**	0.90±0.3	0.86±0.1	0.6	0.83±0.1	0.79±0.1	0.5
**rCBV, mean±SD**	0.78±0.2	0.77±0.2	0.9	0.91±0.1	0.78±0.2	0.04
**rTmax mean±SD**	1.4±1.3	2.1±1.2	0.2	1.1±0.5	2±1.4	0.1

BP-blood pressure, rCBF-relative cerebral blood flow, rCBV-relative cerebral blood volume, rTmax-relative Tmax

## Discussion

This study demonstrates that cerebral perfusion is hypoperfused in the leukoaraiosis regions in acute ICH patients. Although cerebral perfusion is lower in patients with leukoaraiosis, this does not appear to be affected differentially by acute blood pressure reduction, relative to patients without leukoaraiosis.

### Leukoaraiosis and ICH

This study confirms the previous observation that leukoaraiosis is associated with an increased risk of larger admission hematoma volume independent of age.[21] Our finding that ICH volume is larger in patients with leukoaraiosis is also consistent with another recent study in which higher modified Scheltens scale scores (>13) were associated with both larger hematoma volume and expansion.[5] Increased hematoma volume in leukoaraiosis patients is probably multifactorial, including rarefaction of white matter, increased blood brain barrier (BBB) permeability, decreased vascular integrity and/or widening of perivascular spaces.[6,22]

### Leukoaraiosis and cerebral perfusion

The mean rCBF was lower and mean rTmax was delayed in periventricular region with leukoaraiosis. This hypoperfusion was seen across all Fazekas grades. A similar modest decrease in cerebral perfusion within leukoaraiosis regions was also reported in a study of lacunar stroke patients.[23] It has also been demonstrated that MTT is delayed within leukoaraiosis regions of patients without a history of stroke.[10] Our data and these previous studies all suggest that chronic hypoperfusion is associated with leukoaraiosis. However, this hypoperfusion may not be causal.^12^ The patients with leukoaraiosis were older and age is independent predictor of CBF. The reference region white matter CBF was comparable in both groups.

### Leukoaraiosis and ischemia in ICH patients

Recent diffusion-weighted imaging (DWI) MRI studies in patients with primary ICH have demonstrated DWI lesions consistent with ischemic lesions remote from the hematoma. [6,24] Furthermore, these lesions are more common in patients with leukoaraiosis.[25] Microvascular blood flow changes, as demonstrated in the current study, are widespread and likely predispose patients to both ischemia[26] and ICH.[21] The occurrence of these remote DWI lesions may be related to the chronic microvascular pathology itself, rather than an acute hemodynamic response to aggressive BP reduction.

Although cerebrovascular reactivity within leukoaraiosis tissue has been shown to be impaired[10,11], it is not entirely absent. Adequate autoregulation may therefore protect this moderately hypoperfused tissue. While we did not assess autoregulation with serial perfusion measurements in all patients, we did not observe differences in cerebral perfusion in leukoaraiosis tissue, in patients treated with conservative (<180 mmHg) or aggressive (<150 mmHg) blood pressure targets (Table 3).

### Limitations

This study is limited by the small sample size and short-term one time assessment (2h after randomization. This was not pre-specified sub-group analysis. In addition, the majority of our patients had some degree of leukoaraiosis. Regardless, patients were not randomized on the basis of leukoaraiosis, which limits the strength of any conclusions based on our observations. There are baseline differences in the patients with and without leukoaraiosis (age, hematoma volume and BP). This may have affected cerebral perfusion parameters. It is also recognized that CT is less sensitive for leukoaraiosis assessment than MRI. We may therefore have failed to recognize the impact of subtle microvascular changes on perfusion in some patients. Furthermore we excluded cerebral tissue with previous infarcts and perihematoma edema using strict HU criteria.

## Conclusions

BP reduction remains controversial in ICH, but we found no evidence in our study that patients with chronic leukoaraiosis should be treated any differently. This is important, as leukoaraiosis is common in ICH patients, both of which result from small vessel disease.
